# Supplementary material for: Security analysis and secure channel-free certificateless searchable public key authenticated encryption for a cloud-based Internet of things
Source: PLoS One. 2020 Apr 9;15(4):e0230722. doi: 10.1371/journal.pone.0230722 (PMC7144983; doi:10.1371/journal.pone.0230722)
Supplement: Attachment — Submitted filename: Response to Reviewers.doc [file pone.0230722.s001.doc]

**Original Manuscript ID:** PONE-D-19-34208

**Original Article Title: “**Security Analysis and Secure Channel Free Certificateless Searchable Public Key Authenticated Encryption for cloud-based Internet of Things ”

**To:** PLOS ONE Editor

**Re:** Response to reviewers

Dear Editor,

Thank you for allowing a resubmission of our manuscript, with an opportunity to address the reviewers’ comments.

We are uploading (a) our point-by-point response to the comments (below) (response to reviewers), (b) an updated manuscript with yellow highlighting indicating changes, and (c) a clean updated manuscript without highlights (PDF main document), (d) a certificate issued by American Journal Specialist (AJE) to help us with language editing.

Best regards,

Bin Wu, Caifen Wang, Hailong Yao.

**Journal Requirements, Concern # 1:**  Please ensure that your manuscript meets PLOS ONE's style requirements, including those for file naming. The PLOS ONE style templates can be found at http://www.journals.plos.org/plosone/s/file?id=wjVg/PLOSOne_formatting_sample_main_body.pdf and http://www.journals.plos.org/plosone/s/file?id=ba62/PLOSOne_formatting_sample_title_authors_affiliations.pdf.

**Author response:** We have carefully revised our manuscript completely according to the PLOS ONE style templates.

**Author action:** We carefully modified the non-compliant parts of the manuscript according to the style template of PLOS ONE.

**Journal Requirements, Concern # 2:** We suggest you thoroughly copyedit your manuscript for language usage, spelling, and grammar. If you do not know anyone who can help you do this, you may wish to consider employing a professional scientific editing service.

**Author response:** We have carefully revised our manuscript completely according to the suggestion.

**Author action:** We have selected American Journal Experts (AJE) to help us with language editing to ensure that our manuscripts meet journal submission guidelines. This certificate was issued on January 23, 2020 and may be verified on the AJE website using the verification code FFB0-56B0-5224-C035-2CB8 .

**Journal Requirements, Concern # 3:** We note that Figure(s) [1-3] in your submission contain copyrighted images. We require you to either (1) present written permission from the copyright holder to publish these figures specifically under the CC BY 4.0 license, or (2) remove the figures from your submission

**Author response:** We have carefully revised our manuscript completely according to the suggestion.

**Author action:** Since the copyright-protected image material contained in our submitted Figures [1-3] was downloaded from the Internet, it was not easy to obtain permission from the original copyright holder to publish these figures under the CC BY 4.0 license. So we use three paragraphs to describe the meaning of Figure [1-3] in detail respectively , thus remove the figure [1-3].

**Reviewer#1, Concern # 1:** The English writing should be carefully checked before it can be accepted in the Journal.

**Author response:** We have carefully revised our manuscript completely according to the suggestion.

**Author action:** We updated the manuscript by modifying the language usage, spelling, and grammar, following the language editing suggestions provided by American Journal Experts (AJE). This certificate was issued on January 23, 2020 and may be verified on the AJE website using the verification code FFB0-56B0-5224-C035-2CB8 .

**Reviewer#2, Concern # 2:** More related work about cloud-based Internet of Things should be cited in the manuscript.

**Author response:** We have carefully revised our manuscript completely according to the suggestion.

**Author action:** We carefully studied the related work in 2017, 2018 and 2019, selected 6 representative works^[9]-[14]^ from them, introduced them in the introduction, and added them into the reference.

***REFERENCES:***

*[9] Babu S M, Lakshmi A J, Rao B T et al. A study on cloud based Internet of Things: CloudIoT. In: Proc. GCCT 2015. 60-65. doi:10.1109/GCCT.2015.7342624.*

*[10] Conti M, Dehghantanhab A, Frankec K, Watsond S. Internet of Things security and forensics: Challenges and opportunities. Future Generation Computer Systems. 2018, 78:544-546. doi: 10.1016/j.future.2017.07.060.*

*[11] Ojha T, Misra S, Raghuwanshi R N, Poddar H. DVSP: Dynamic Virtual Sensor Provisioning in Sensor Cloud-Based Internet of Things. IEEE Internet of Things Journal. 2019, 6(3):5265-5272. doi: 10.1109/JIOT.2019.2899949.*

*[12] Pan W, Chai C. Structure-aware Mashup service Clustering for cloud-based Internet of Things using genetic algorithm based clustering algorithm. Future Generation Computer Systems. 2018, 87:267-277. doi:10.1016/j.future.2018.04.052.*

*[13] Meerja K A, Naidu P V, Kalva S R K. Price Versus Performance of Big Data Analysis for Cloud Based Internet of Things Networks. Mobile Netw Appl. 2019, 24:1078-1094. doi: 10.1007/s11036-018-1063-6.*

*[14] Boveiri H R, Khayami R, Elhoseny M et al. An efficient Swarm-Intelligence approach for task scheduling in cloud-based internet of things applications. J Ambient Intell Human Comput. 2019, 10:3469-3479. doi: 10.1007/s12652-018-1071-1.*
